# Addressing the Challenges in Using Synthetic Data for Health Research: Application to Cardiology

**DOI:** 10.2196/92930

**Published:** 2026-07-31

**Authors:** Louise Baschet, Sebastien Marque, Stéphane Locret, Jens Jenssen, Vanessa Barbet, Patrick Jourdain

**Affiliations:** 1 Horiana, 80 bis rue Paul Camelle, Bordeaux, 33100, France, 33 687270347; 2 Research Department, Ramsay Santé, Paris, France; 3 Department of Clinical Science and Education, Södersjukhuset, Karolinska Institutet and Capio St Görans Hospital, Stockholm, Sweden; 4 Medical Department, Ramsay Sante, Paris Saclay University, Paris, France

**Keywords:** health care, machine learning, synthetic data, virtual patients, cardiology, medical research

## Abstract

Synthetic data offer significant potential for cardiology research by enabling data sharing, preserving privacy, and supporting machine learning model development. By generating artificial patient records that reflect real-world distributions, synthetic data can accelerate clinical research, improve model performance for rare cardiovascular conditions, and facilitate transnational collaborations that would otherwise be restricted by data-sharing barriers. Despite these advantages, the increasing use of synthetic data raises important ethical, regulatory, and methodological concerns that remain insufficiently addressed. Key challenges include assessing the validity and generalizability of synthetic datasets, understanding their limitations in representing complex and heterogeneous patient populations, and preventing the amplification of existing biases in cardiovascular care. Current regulatory frameworks, including the General Data Protection Regulation (GDPR) and Health Insurance Portability and Accountability Act (HIPAA), do not fully address emerging risks such as reidentification and data leakage, and there is no harmonized guidance to govern the use of synthetic data as stand-alone evidence for medical device evaluation or therapeutic research. In this viewpoint, we argue that responsible integration of synthetic data in cardiology requires, first, clear differentiation between synthetic data as a privacy-preserving distributional substitute and synthetic data as a counterfactual simulation tool, and, second, fit-for-purpose governance frameworks that pair rigorous utility and fidelity testing with explicit, adversary-aware privacy evaluation before synthetic cohorts are accepted as evidence in research or product evaluation. A prerequisite for that governance is conceptual clarity about what synthetic data are being used for. Synthetic data in health care serve 2 fundamentally distinct roles that carry entirely different validity requirements, failure modes, and regulatory implications, yet they are routinely conflated. The first role is as a privacy-preserving distributional substitute: the goal is statistical fidelity to the real data distribution, so that analyses of the synthetic dataset yield results equivalent to those of the original. The second role is as a tool for counterfactual simulation: the goal is to generate data that could not have been observed, such as rare conditions, hypothetical interventions, or extrapolations to new populations. These 2 roles are methodologically distinct. A dataset that accurately reflects real-world distributions may be inadequate for extrapolating findings to underrepresented subgroups. Conversely, a simulator optimized for novel scenario generation may systematically diverge from real-world distributions. This distinction informs every subsequent discussion of validity, bias, and regulation in this viewpoint and our proposed 4 concrete actions for the cardiology research community, including mandatory 3-layer (fidelity, utility, and privacy) validation, systematic subgroup reporting, explicit intended-use scoping, and domain-specific acceptability thresholds for synthetic data–based evidence.

## Introduction: Synthetic Data as an Emerging Tool in Cardiology

Used for many decades in other fields, synthetic data were adopted more recently in health care and have been shown to offer significant advancements. Specifically, synthetic data address challenges related to data privacy, progressively optimize care, and improve clinical research [1-3]. In oncology, models trained on synthetic data have been shown to predict survival more accurately than models trained on original data [4]. In addition, use of massive data can accelerate research, reducing costs and inclusion periods associated with clinical trials and data analysis. However, while the benefits are now becoming well known, the limitations, especially legal framework–related limitations, ethics, and responsibilities, are less well known [5-7]. In this context of growth, understanding the relevance of synthetic data, their compliance with regulations, and their potential to address the lack of patient data in various scenarios is essential.

In this viewpoint, we provide an overview of the main applications and roles of synthetic data in cardiology, highlight the conceptual distinction between distributional and counterfactual uses, and examine the associated methodological, ethical, and regulatory challenges. We begin by providing definitions for the key terms used in the literature, and we outline the methods, benefits, and challenges of using synthetic data in cardiology research. Our central argument is that synthetic data in cardiology should not be viewed merely as a privacy-preserving technical workaround, but as a methodologically distinct form of evidence that requires dedicated governance, validation standards, and interpretability safeguards. The absence of such standards is itself a major risk: without them, synthetic data risk being adopted inconsistently, reinforcing the blind spots they are meant to address. We conclude by underlining the potential impact of synthetic data for transnational research use without compromising patient rights to privacy and by proposing 4 concrete actions for the cardiology research community to operationalize this governance agenda.

Cardiology is a particularly important test case for this argument. The high-frequency, time-series nature of key cardiac signals (electrocardiograms [ECGs], photoplethysmogram [PPG], and phonocardiograms) imposes strict fidelity requirements on generative models that are not encountered in the same way in other medical specialties. The spatial complexity of cardiac imaging modalities (magnetic resonance imaging [MRI] and computed tomography [CT]) demands higher-dimensional generative capacity, and the prevalence of rare arrhythmias and structural anomalies create chronic data scarcity for training predictive models. Additionally, the long-term disease trajectories, characteristic of chronic heart failure and coronary artery disease, challenge generative models that capture cross-sectional distributions but cannot capture longitudinal dynamics. These domain-specific constraints make the governance agenda outlined in this viewpoint particularly relevant and timely.

## Clarifying the Landscape: Key Terms in Synthetic and Virtual Patient Data

### Synthetic Data

The seminal work on synthetic data by the Royal Society and The Alan Turing Institute stresses functional and intentional aspects of synthetic data that have “been generated using a purpose-built mathematical model or algorithm, with the aim of solving a (set of) data science task(s)” [8]. Synthetic data is artificial data generated from original real data and a model that is trained to reproduce the characteristics and structure of the original data [9]. Synthetic and original databases should deliver very similar results when subjected to the same statistical analysis despite different cell values. The accuracy of synthetic data as a proxy for the original data reflects the *utility* of the method and the model.

### Data Augmentation

In computer vision and signal processing, augmentation typically involves transforming existing data (eg, rotation, scaling, and noise addition), while preserving labels [10]. For structured clinical or electronic health record data, augmentation more often relies on generating new patient records using statistical or machine learning (ML) models trained on real cohorts, resulting in datasets that mix real and synthetic patients [11].

### Virtual Patients

Virtual patients are simulated patients used in medical training, providing a risk-free environment for health care professionals to refine their skills [6,12]. For example, a simulator was developed for patients living with heart failure to improve care delivery. It combines comprehensive hemodynamic and energetic data for personalized prognostication, such as identifying the need for heart rhythm therapy and forecasting response to device and medical therapies [13]. This is particularly useful for patients with complex clinical situations and/or drugs with a narrow therapeutic margin. However, the model can only reproduce existing elements and cannot identify new sources of interactions.

### Synthetic Patients

Synthetic patients mimic real patient characteristics without using actual patient information. They are generated by statistical methods or AI and are represented in datasets, primarily for privacy-preserving research purposes [11].

### Digital or Virtual Twins

Digital or virtual twins are real-time virtual replicas of physical or biological systems used to simulate patient behaviors and optimize treatments in cardiology [6,14]. These twins receive continuous updates of multiple data types to predict disease progression. Typically, twinning is partial to preserve privacy. Current innovation is increasing in cardiovascular digital twin research [15], including growing industry and academic patent activity [6,14]. Digital twins could also help plan complex surgeries, rhythm-based procedures, or to train junior cardiologists.

Taken together, these three categories exist on a spectrum: (1) virtual patients are mechanistic simulation constructs primarily used for training and procedural planning, without direct derivation from a specific individual’s records; (2) synthetic patients are population-level statistical surrogates generated to preserve privacy or augment data; and (3) digital twins are individual-level, continuously updated dynamic models. Understanding this spectrum is essential, as each category carries different validity requirements, failure modes, and regulatory implications.

## How Synthetic Data Is Generated: Methods and Underlying Models

Artificial data mimicking real patient statistics can be generated from 3 sources: [11] by creating new data points similar to one patient, by modifying data from one patient using the nearest neighbors, or by characterizing the patient population globally to create synthetic patients representing shared traits. Focusing on the last global approach, synthetic data generation can use multiple algorithms [5]. Two types of models are described in McDuff et al’s review [16].

First, physical models require detailed parameterization, offering interpretability and known constraints, making them a safe choice for data generation. However, they tend to be task-specific, may not generalize well across domains, and often demand significant computational resources. Complex real-world systems can lead to model mismatches, with unknown factors posing risks to practical use.

Second, statistical generative models, such as generative adversarial networks (GANs) [17] and variational autoencoders (VAEs) [18], replicate dataset distributions and can produce realistic, high-resolution data. These models are generally easier to create than physical simulators but may lack interpretability and control. Their reliability in medical applications heavily depends on the quality of the original data (used for training) and the use of supervised learning for better regulation of output [16].

These models use explicit or implicit data generation methods [11]. Explicit approaches are used when the relationship between the variables is known or at least assumed. In these explicit approaches, it is possible to interpret the strength of association between variables (eg, covariates, outcomes, or interactions).

The first and most frequent explicit approach is mechanistic models, which are typically based on systems of ordinary or partial differential equations describing physiological processes and their interactions over time under known constraints. The second explicit approach is Bayesian networks, which can be used to generate new synthetic data through probabilistic graphical models that represent knowledge about variables and their dependencies. Bayesian networks can be used to make inferences about specific variables of interest in the network, depending on the other variables in the network.

Implicit approaches are used where there is no need to know how the variables are linked, as the model will determine this by itself, and there is no need to interpret the structure of the model. GANs and VAEs, and denoising diffusion models are all implicit approaches. GAN-based methods have significantly enhanced data analysis capabilities [11]. In health care, GANs have been successfully applied to diverse imaging modalities in cardiology, such as MRI and CT, yielding promising results [19-21]. However, a notable limitation is that most studies have relied on large training datasets or low-dimensional data. While initially less popular than GANs for data augmentation, VAEs are gaining traction in medical applications, particularly for classification and segmentation tasks [22,23]. The main limitation of VAEs is that they can produce imprecise samples when not properly tuned, especially with limited training data. Diffusion models represent an emerging class with promising fidelity for imaging tasks but remain limited in cardiology-specific applications.

To link this taxonomy to application, physical and mechanistic models are preferred when physiological interpretability and regulatory transparency are required (eg, hemodynamic simulation for device testing), at the cost of generalizability. GANs are well-suited for high-dimensional imaging data but are particularly prone to bias amplification when training datasets are small or demographically skewed. VAEs are better suited to lower-dimensional structured data and classification tasks. Bayesian networks are appropriate when variable relationships are partially known and interpretability is a priority. Choosing the right model for the intended use case is a prerequisite for the validation process described below.

## Applications and Benefits for Cardiology Research

### Overview

Synthetic data enables ML with large, labeled datasets that are otherwise difficult or expensive to obtain due to privacy or access constraints. Synthetic data also accelerates software testing and model pretraining and is widely used in health care to support quicker, smaller trials with fewer privacy risks. For example, synthetic data generation can reduce the time required to set up a cardiovascular data platform. In 1 reported case, synthetic data generation reduced the time required to set up a cardiovascular data platform from 1.5 years to 1 month [24], illustrating the potential for efficiency gains, though systematic evidence on typical effect sizes and the conditions under which such benefits are realized remains limited. AI models trained with synthetic or mixed data (real and synthetic data) often match or outperform those trained with real data only [25]. For example, when cancer survival prediction models were trained on synthetic data, they showed improved performance of >0.1% and, for extremely small datasets, outperformed those using only original datasets [4].

Synthetic data can significantly enhance the predictive power of AI models and optimize clinical research in cardiology through several key mechanisms.

### Augmenting Data Volume and Simplifying Exportability of Health Care Datasets

Synthetic data can bolster the volume of available data, enabling more robust and comprehensive analyses [8,26]. This is particularly valuable in cardiology, where large datasets are crucial for training accurate predictive models. However, this benefit is conditional: augmenting data volume only improves downstream model performance when the generative model has successfully captured the true underlying distribution of the real data, a condition that must be verified through explicit utility and fidelity testing before the synthetic dataset is used.

### Predicting Clinical Trial Results

In silico trials represent an experimental application used to expand testing methods. For example, in silico trials enable results to be extrapolated to populations with different demographics or characteristics [27], facilitating the inclusion of additional comparators, and they enable the extension of short-term study results to long-term projections [28].

### Addressing Data Scarcity

When real patient data are limited, synthetic data can fill gaps and improve performance of predictive models. Synthetic data can be used to simulate rare cardiovascular conditions, allowing AI models to learn from a larger number of cases than would be available in real-world datasets [29]. For rare events, simulators can be used to create synthetic examples and thereby reduce the lack of sensitivity of a model trained with class imbalance. Examples include detecting arrhythmia from PPG or ECG [16,26].

### Enhancing Data Diversity

When intentionally designed with diversity objectives and appropriate constraints, synthetic data can introduce greater variability into datasets [6]. However, this benefit is not automatic. Generative models trained on biased data will reproduce those biases. Achieving genuine diversity enhancement requires deliberate methodological choices, including stratified sampling, conditional generation targeting underrepresented groups, and rigorous subgroup validation.

### Preserving Patient Privacy

By using synthetic data generated from real patient records, researchers can develop and test AI models without compromising sensitive health care–related patient information [6,24].

### Improved Accuracy of Diagnosis

Synthetic patient data have been widely adopted for the processing of physiological signals and downstream classification in physiological measurement [16]. In particular, researchers have investigated the synthesis of cardiac signals, namely ECGs, phonocardiograms, and PPG [16]. Models trained with real ECGs augmented with synthetic ECGs have shown improved accuracy of arrhythmia diagnosis [30].

## Challenges and Limitations in Applying Synthetic Data to Cardiology

**Figure 1.**
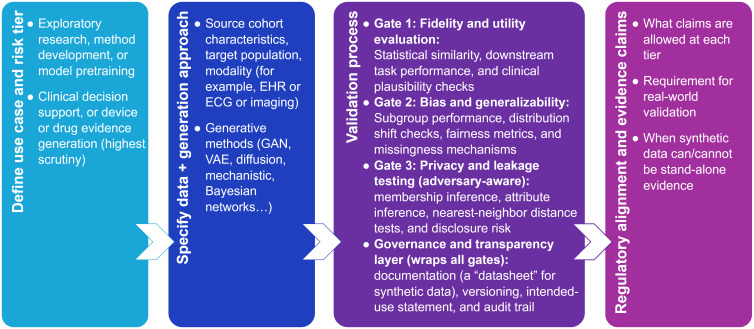
Proposed fit-for-purpose workflow for the evaluation and governance of synthetic cohorts. ECG: electrocardiogram; EHR: electronic health record; GAN: generative adversarial network; VAE: variational autoencoder.

While synthetic patients offer promising avenues for research and clinical applications, Figure 1 proposes a fit-for-purpose evaluation and governance workflow that links technical performance (utility and fidelity), generalizability and bias, privacy risk, and regulatory expectations to the intended use of a synthetic cohort in cardiology.

### Data Validity and Reliability (Fidelity)

One of the primary concerns with synthetic data is their validity. If the algorithms used to generate synthetic data do not accurately reflect the complexities of real-world patient populations, the conclusions drawn from studies using these data may be misleading. In cardiology, where patient responses can vary significantly, this is a critical issue. Addressing this systematically requires 3 complementary validation layers, as reflected in the governance workflow shown in Figure 1. First, fidelity validation involves assessing statistical similarity between synthetic and real distributions, covering both marginal and joint distributions and, for cardiology, domain-specific signal properties such as QRS morphology and RR-interval dynamics in ECG data. Second, utility validation involves testing whether models trained on synthetic data perform equivalently to those trained on real data for the downstream task of interest, using a “train-on-synthetic, test-on-real” paradigm. Third, privacy validation applies adversary-aware tests, including membership inference attacks (which test whether an adversary can identify individuals from the training set) and attribute inference evaluation (which tests whether sensitive attributes can be reconstructed), rather than relying on the absence of direct identifiers alone. All 3 layers should be treated as minimum reporting standards for any cardiology study using synthetic data.

### Generalizability

Synthetic data may not generalize well to diverse or to highly specific populations [16]. Cardiology encompasses a wide range of conditions and patient demographics, and synthetic models may fail to capture the nuances of different patient groups, leading to biased results. Learned generative models produce statistics relative to the underlying data distribution, and large-parameter models have been found to amplify bias [31]. AI or ML models extract or adapt their parameters from training data, making them vulnerable to bias [32].

### Ethical Considerations

The use of synthetic data raises ethical questions, particularly regarding consent and the representation of marginalized populations. Ensuring that synthetic data does not perpetuate existing biases in health care is essential for ethical research practices [7,33].

### Integration With Clinical Practice

Integrating synthetic patient models into clinical workflows poses challenges. Health care providers may be hesitant to rely on models that are not validated against real-world outcomes, which can hinder the adoption of these technologies in practice.

### Regulatory Blind Spots

There are currently no harmonized recommendations from agencies or regulatory authorities defining criteria for the acceptability of synthetic patient cohorts for the evaluation of health products or medical devices, and the very concept of using synthetic patient cohorts for this purpose remains without consensus [7,9,11,34,35]. By providing realistic datasets without exposing sensitive patient information, synthetic data enables health care organizations to comply with privacy regulations, such as the Health Insurance Portability and Accountability Act (HIPAA) and the General Data Protection Regulation (GDPR); however, there is still no clear legislation specifically governing the use of synthetic data in health care. Current data protection regulations are limited in their ability to address all the potential risks associated with synthetic data, such as reidentification or data leakage [34,36]. The rise in the use of synthetic data has created an expanding industry of companies offering synthetic data solutions and enabling cross-border data sharing, sometimes beyond the confines of traditional data protection legislation. Regulatory agencies are actively exploring the use of synthetic data, but as of 2025, synthetic data is not yet broadly trusted as stand-alone evidence for regulatory approval, and findings derived from synthetic data are required to be validated on real data.

Existing partial frameworks offer some orientation but leave critical gaps. The European Medicines Agency’s 2024 *Reflection Paper on the Use of Artificial Intelligence (AI) in the medicinal product lifecycle* [32] acknowledges simulation and synthetic data as emerging tools but stops short of defining acceptability criteria for their use as stand-alone evidence. The Food and Drug Administration’s evolving guidance on AI and ML-based software as a medical device similarly does not yet address synthetic patient cohorts as a distinct evidence category [37]. Any future harmonized framework would need to specify at minimum: (1) the intended-use scope within which a synthetic dataset has been validated and may legitimately be used, (2) minimum fidelity thresholds appropriate to the application risk tier, (3) mandatory adversary-aware privacy testing before regulatory submission, and (4) a requirement that findings derived from synthetic data be validated on real-world data before supporting regulatory decisions. Until such a framework exists, researchers and sponsors should proactively document their datasets against these criteria to support transparency and anticipate future expectations. Recent work underscores that the core barrier is not only regulatory uncertainty but also the lack of agreed, quantitative ways to demonstrate that a synthetic dataset is both useful and safe. In their scoping review, Kaabachi et al [5] identified dozens of distinct approaches to assess synthetic data utility and only a small set of approaches used to evaluate privacy risk, concluding that many studies do not assess privacy protection at all and that, when they do, residual risks are often underestimated. This supports the call for harmonized evaluation frameworks that pair utility metrics with explicit, adversary-aware privacy testing before synthetic cohorts are considered acceptable evidence in cardiology research or product evaluation.

### Privacy

Although synthetic data theoretically offer patient privacy protection, there remains a risk that synthetic data generation may leak personal information [8,16]. Strictly, it is not possible to determine whether synthetic data are sufficiently different from the original samples to be considered anonymized [36]. There are no robust and objective methods of determining whether a synthetic dataset is sufficiently different from the original real dataset to be classified as truly anonymous, and recent reviews highlight inconsistent practice and the frequent absence of privacy-risk evaluation in published synthetic data studies [5]. This is a fundamental, not peripheral, problem: without a reliable method to certify sufficient dissimilarity from training data, the use of synthetic datasets in research settings remains legally ambiguous under both GDPR and HIPAA, regardless of scientific utility. In practice, privacy assurance requires adversary-aware testing rather than reliance on the absence of direct identifiers. Two tests are particularly relevant: membership inference attacks, which evaluate whether an adversary can determine that a specific individual’s record was used in training; and attribute inference evaluation, which tests whether sensitive attributes (such as diagnosis or treatment history) can be reconstructed from synthetic records. Kaabachi et al [5] found that most published synthetic data studies do not perform privacy-risk evaluation at all, and that when they do, adversarial attack methods are rarely applied and residual risks are frequently underestimated. The cardiology field should treat adversary-aware privacy validation as a nonnegotiable component of any synthetic data reporting standard, consistent with the privacy gate in Figure 1.

### Technical Limitations

Algorithms used to generate synthetic data can have limitations in terms of complexity and accuracy. In cardiology, where patient data can be highly variable and influenced by numerous factors, these limitations can impact the effectiveness of synthetic data for modeling patient outcomes.

## Conclusions

Overall, while synthetic patient research and synthetic data hold substantial promise for advancing cardiovascular research, careful consideration of the associated challenges is necessary to ensure that these tools are used effectively in clinical practice. Synthetic datasets and in silico studies could transform the future of cardiovascular research, allowing the use and transfer of reliable datasets without compromising patient privacy and addressing data scarcity. Realizing this potential depends on recognizing and governing the 2 fundamentally different roles that synthetic data can play, either as a privacy-preserving distributional substitute for existing data or as a counterfactual simulator for rare events and hypothetical scenarios, and on applying validation criteria appropriate to each role. Within strict boundaries and under specific conditions to limit potential bias, synthetic data can uphold privacy, equity, and safety, but care must be taken to avoid introducing flaws, blind spots, and propagating or exaggerating biases. Synthetic datasets represent a fundamentally different form of evidence, and their value depends on how rigorously their limitations, sources of bias, and intended uses are defined and governed. In the absence of shared methodological standards and explicit regulatory expectations, synthetic data risks being adopted inconsistently, potentially reinforcing blind spots rather than mitigating them. We argue that progress in this field now requires a collective shift from exploring what synthetic data *can* do to agreeing on when, how, and for which purposes they *should* be used in cardiology research. Within clearly defined boundaries and under transparent validation and governance conditions, synthetic data can support innovation, data sharing, and equity without compromising scientific credibility or patient trust. Importantly, the challenges associated with synthetic data are amplified in cardiology, where clinically relevant information is often encoded in high-frequency signals, complex cardiac anatomy, and long-term disease trajectories.

To operationalize this governance agenda, we propose 4 concrete actions for the cardiology research community. First, mandatory three-layer validation for any publication using synthetic cardiology data: (1) fidelity validation (statistical similarity, including signal-specific properties such as QRS morphology for ECG data), (2) utility validation (train-on-synthetic, test-on-real performance equivalence for the downstream task), and (3) privacy validation through adversary-aware testing, including membership inference and attribute inference evaluation. Second, mandatory subgroup reporting requires performance metrics should be disaggregated by relevant demographic subgroups, rather than reported only at the population level. Third, intended-use scoping dictating that any synthetic dataset should document the specific purpose for which it was validated, consistent with the fit-for-purpose principle illustrated in Figure 1. Fourth, engagement by cardiology societies and regulatory agencies to define domain-specific acceptability thresholds for synthetic evidence, beginning with lower-stakes applications such as model pretraining and simulation before moving to higher-stakes regulatory submissions.

## Abbreviations

CT: computed tomography
ECG: electrocardiogram
GAN: generative adversarial network
GDPR: General Data Protection Regulation
HIPAA: Health Insurance Portability and Accountability Act
ML: machine learning
MRI: magnetic resonance imaging
PPG: photoplethysmogram
VAE: variational autoencoder
